# Selection of Durable, Environmentally Friendly, and Cost-Effective Asphalt Mixtures

**DOI:** 10.3390/ma15144873

**Published:** 2022-07-13

**Authors:** Vikas Kumar, Erdem Coleri, Ihsan Obaid, Anda Ligia Belc, Alex James Sutherland

**Affiliations:** 1 School of Civil and Construction Engineering, Oregon State University, Corvallis, OR 97331, USA; erdem.coleri@oregonstate.edu (E.C.); ihsan.obaid@qu.edu.iq (I.O.); suthalex@oregonstate.edu (A.J.S.); 2 Road and Transport Department, College of Engineering, University of Al-Qadisiyah, Al Diwaniyah 58002, Iraq; 3 Faculty of Civil Engineering, Politehnica University Timisoara, 300006 Timisoara, Romania; anda.belc@student.upt.ro

**Keywords:** volumetric mix design, balanced mix design, asphalt density, warm mix asphalt, life cycle assessment, cracking, rutting, life cycle cost analysis

## Abstract

In recent years, due to the advent of several additives and innovations, asphalt mix design has become more complex. The mixes meeting the volumetric mix design requirements may still fail prematurely in the field. Thus, a transition from a simplistic volumetric-based mix design to a performance-based mix design is required, which was also envisioned in the Strategic Highway Research Program (SHRP) and Superpave mix design. In addition to performance verification, asphalt mix designs should also be evaluated for the life-cycle costs and environmental impact to encourage durable as well as sustainable and cost-effective alternatives. In this study, three asphalt mixtures with different reclaimed asphalt pavement (RAP) contents and additives were evaluated for cracking and rutting performance by using different performance thresholds for asphalt mixtures that are generally used in the construction of high-volume roads in Oregon. A balanced mix design process was followed to determine the required binder content for the three mixtures. Based on the life cycle cost and environmental impact analyses, the mixture with warm mix additive (WMA) was selected as the most economically and environmentally viable asphalt mixture to be used for construction in Oregon.

## 1. Introduction

Asphalt mix design, at its core, is a simple process of blending aggregates (of different sizes), asphalt binder, and, in most cases now, recycled asphalt materials (RAMs). The mix obtained from this design process is expected to meet the specifications established by the state departments of transportation (DOTs) or government agencies. In recent years, due to the advent of several additives and innovations, asphalt mix design has become more complex. Moreover, the mixes, despite meeting the volumetric requirements, may sometimes fail prematurely in the field. For example, the volumetric design results in a lower binder content in asphalt mixes in Oregon, making them susceptible to cracking prematurely. Oregon Department of Transportation’s (ODOT’s) Pavement Management System has shown that asphalt mixes placed in the last 20 years have had a tendency to develop premature cracking after 6 to 8 years of service before reaching the structural design life of 15 years. This happens because the specifications were established based on the assumption that there is a strong empirical correlation between the volumetric parameters of mix design, such as air void content, voids in mineral aggregates (VMAs), voids filled with asphalt (VFAs), and the field performance of asphalt pavements. However, this correlation is generally valid when there are no special additives or technologies used for the production of asphalt. As such, the use of new additives and strategies, such as Reclaimed Asphalt Pavement (RAP), warm mix asphalt (WMA), and polymer-modified asphalt binders (PMA), in the asphalt mix design may complicate the asphalt mix design process and result in unreliable field performance prediction. Thus, a transition from a simplistic volumetric-based mix design to a performance-based mix design is required, which was also envisioned in the Strategic Highway Research Program (SHRP) and the Superpave mix design [1]. Many researchers, DOTs, and government agencies have advocated this transition, and several studies have been conducted to explore the integration of performance verification in the current mix design process [2,3,4,5,6,7,8,9,10,11].

In addition to performance verification, asphalt mix designs should also be evaluated for the life-cycle cost and environmental impact to encourage durable as well as sustainable alternatives. Among several strategies, three promising strategies that have gained the attention of researchers and agencies alike are the (1) increased in-place density, (2) high percentage of RAP in the mix, and (3) use of WMA. Increased in-place pavement density or reduced air voids have been found to significantly improve both the cracking and rutting performance of asphalt pavements [12,13,14,15,16]. In addition, improved performance can lead to long-term cost savings based on the life cycle cost analysis (LCCA) of asphalt pavements [15]. The use of high RAP/RAS in hot mix asphalt (HMA) mixes has the potential of not only creating direct cost savings but also conserving natural resources and promoting sustainable practices in the asphalt pavement industry by incentivizing recycling. Although the average maximum allowable RAP is fixed at 30% by many state DOTs in the U.S.A. [17], past studies have indicated that this limit can be pushed to 50% [18] or even 100% RAP [19]. Despite the aforementioned benefits, high-RAP mixtures must be evaluated for performance before use in the field because of the concerns related to long-term durability [20,21].

Similar to high-density and high-RAP mixes, the use of WMA in asphalt pavements has numerous advantages over conventional HMA mixes. According to [15,22], WMA yields pavements of comparable or higher performance quality at lower production temperatures than the standard HMA mix. WMA also provides additional benefits of enhanced compaction [15] and reduced energy consumption [23].

In Oregon, fatigue cracking is the major distress mode for asphalt concrete pavement structures. It is one of the main reasons for large road maintenance and rehabilitation expenditures, as well as reduced user comfort and increased fuel consumption due to high road roughness. Increasing the asphalt binder content, using modified binders, and/or using softer binder grades were proved to improve fatigue cracking resistance [24,25]. Coleri et al. [24] showed that the binder content of the asphalt mixtures produced with the current volumetric design method can be increased without having rutting failures. The low binder content suggested by the current volumetric design methods results in early fatigue cracking and moisture damage. To address these issues, Coleri et al. [11] developed a robust performance-based asphalt mix design method to be able to recommend effective strategies for performance improvement. In this study, balanced mix design (BMD) procedures developed by Coleri et al. [11] in the SPR801 ODOT research project were followed to design three asphalt mixtures for Oregon roads with high traffic levels (Level 4 mixtures). Balanced mix design is currently a hot topic in the United States. Many studies are focused on the performance verification of the asphalt mixes as well as life cycle cost analysis (LCCA). However, no study has incorporated life cycle assessment (LCA) or environmental impact along with a balanced mix design method and LCCA in the asphalt mix selection process. The objectives of this study are to:

- Design three asphalt mixtures with different constituents to determine the most effective strategies for road construction;
- Evaluate the trial mixes for cracking and rutting performances;
- Determine the design binder content range for each mix using a balanced asphalt mix design method developed for Oregon by incorporating performance tests for rutting and cracking into the current volumetric design process [11];
- Determine the cost and environmental impact of all three mixtures by performing life cycle cost and environmental impact analysis;
- Recommend the “best” asphalt mixture for the given conditions by considering the cost-effectiveness, environmental impact, and long-term performance of the mixes.

## 2. Materials and Methods

### 2.1. Materials

This section provides information about the materials used in this study (including virgin binders, virgin aggregates, and RAP materials). The materials were sampled from an asphalt plant located near Tigard, Oregon. The samples were produced at Oregon State University Asphalt Materials and Pavements (OSU-AMaP) Laboratory from the raw materials that were used in the actual field construction project.

Three different asphalt mixtures were used in this study (called Mix1, Mix2, and Mix3). These mixes varied in gradation, amount of RAP content, and presence of additives. Mix1 was further divided into two mixes, Mix1_AV5 and Mix1_AV7, differing by the compacted air void contents of the test samples (5% and 7%, respectively) to quantify the impact of density on performance (both designed by the Superpave mix design method via Superpave Gyratory Compactor (SGC)). Mix2 had 45% RAP content, and Mix3 was identical to Mix1 except that in Mix3, a chemical warm-mix additive was used. Both Mix2 and Mix3 were compacted to 93-percent of the theoretical maximum density (±0.5%) in a gyratory compactor to produce test samples with a conventional 7% air-void content. In this study, BMD samples were produced with 7% air-void content as a 93% density during construction is the expected average density for contractors in Oregon.

**Figure 1:**
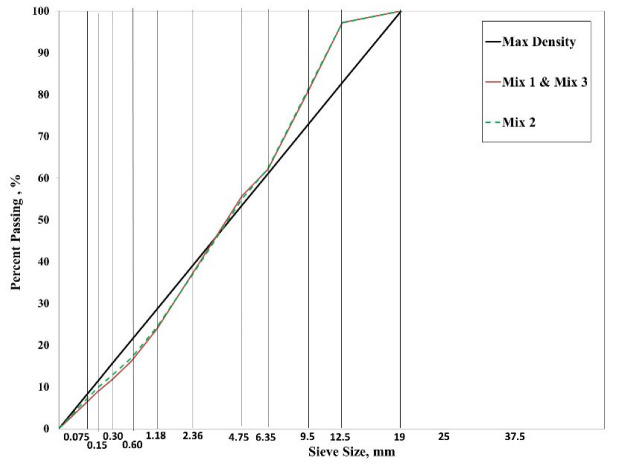
Gradation curves for all three mixes on a 0.45 power chart.

Figure 1 shows the gradation curves used for the production of the three mixtures. These gradations were selected based on the positive past experiences of the Oregon Department of Transportation (ODOT). Once the target gradation was finalized, three trial binder contents were selected for the mix design. For each binder content, theoretical maximum specific gravity (G_mm_) samples were mixed in triplicate according to AASHTO T 312-12 [26] and their respective G_mm_ values were determined as per AASHTO T 209-12 procedures. Subsequently, three replicate mix design samples were prepared for each binder content and compacted in the gyratory compactor.

The binder content corresponding to the target design air void was selected as the optimum binder content (OBC) for each mix. The volumetrics and the other mix design variables of the three mixes used in this study are summarized in Table 1. The asphalt mixture with 45% RAP has a lower binder content than the other two mixes based on the results of the volumetric mix design. This is expected to be a result of the fine gradation with higher dust content used to prepare those mixes (Figure 1). It should be noted that Mix3 with the WMA additive was not volumetrically designed. The binder content suggested by the volumetric design for Mix1 was used to prepare asphalt mix test samples for Mix3. The reason was to clearly identify the impact of using WMA additives on cracking and rutting resistance.

It should be noted that RAP and hydrated lime are added by weight of the mix, whereas WMA is added by weight of the asphalt binder. Hydrated lime is an inorganic compound, whereas the chemical WMA has polymeric content. Dust-to-binder ratios for all asphalt mixtures are within the limits required by the ODOT (0.8–1.6).

### 2.2. Preparation of Laboratory Mixed-Laboratory Compacted (LMLC) Specimens

The AASHTO T 312-12 [26] specification was used for mixing and compaction of the samples to achieve the target gradation and the target air voids (7 ± 0.5%) for all the mixes (except Mix1_AV5 for which the target air content was 5 ± 0.5% to determine the impact of density on performance). Prior to mixing, the aggregates and the binder were heated separately in the oven for 2 h. The binder was heated at the mixing temperature (173 °C), virgin aggregates were heated at 10 °C higher than the mixing temperature, and RAP was heated at 110 °C. After mixing, the loose mixtures were conditioned in the oven for 2 h at 135 °C based on the findings of previous research studies [11,27] to simulate short-term aging (STA). These short-term-aged loose mixtures were further conditioned at 95 °C for 24 h to simulate long-term aging (LTA) according to the LTA protocol for the Oregon mixtures [11,28]. Only the Semi-Circular Bend (SCB) test samples were subjected to LTA conditioning. After STA and LTA conditioning, mixtures were further kept in the oven at compaction temperature (160 °C) for 2 more hours prior to compaction in the SGC.

For warm mix asphalt sample preparation, aggregates and RAP were batched following the same guidelines as the hot mix asphalt. Before mixing, the binder and the chemical warm mix additive were mixed using a stationary countertop mixer. The chemical additive dosage was calculated according to Equation (1) [29], considering the total binder in the mix (virgin binder and binder derived from RAP) and starting from a target additive dosage (in this case, it was considered 0.5% by weight of total binder). For warm mix asphalt, the mixing temperature of 140 °C and the compaction temperature of 126 °C were adopted based on the manufacturer’s recommendation [29]. The STA and LTA conditioning methods were kept the same as HMA mix.

% Adjusted Chemical Additive Dosage = [(% Target Additive dosage) × (% Total binder)]/(% Total binder − % Binder from RAP)(1)

### 2.3. Test Methods

To evaluate the cracking and rutting performance of the asphalt mixture samples prepared in the study, SCB and Hamburg Wheel Tracking Test (HWTT) methods were followed, respectively. The AASHTO T 324 [30] standard was followed for the HWTT testing. As per the standard, either a slab or a cylindrical specimen can be tested. Tests are conducted by immersing the asphalt concrete sample in a hot water bath (at 40 °C or 50 °C) and rolling a steel wheel across the surface of the sample to simulate vehicular loading. Approximately 20,000-wheel passes are commonly used to evaluate the rutting and stripping resistance of a sample. The test provides information related to the total rut depth, post-compaction, creep slope, stripping inflection point, and stripping slope of the asphalt concrete sample. In this study, rut depth after 20,000-wheel passes is used for rutting performance evaluation. 

In a previous research study performed at Oregon State University, the semi-circular bend (SCB) test was selected as the most effective cracking experiment to characterize asphalt mixtures used in Oregon [24]. Therefore, SCB tests were conducted in this study to determine the cracking resistance of asphalt mixtures and to determine a suitable threshold for the test’s output parameter (flexibility index) to be used as an acceptance criterion in the proposed balanced asphalt mixture design process. A test method developed by Ozer et al. [31] was adopted for SCB testing with the displacement rate modified to 0.5 mm/min from the originally suggested rate of 50 mm/min for improved accuracy and less variability in results [16,24].

The fatigue cracking test (SCB) was conducted at an intermediate temperature (25 °C) when the mix was neither too soft nor too brittle. The rutting test (HWTT) was investigated at a hotter temperature of 50 °C at which the mix was soft.

### 2.4. Experimental Design

This study was performed to evaluate three different mixes for their cracking and rutting performance and volumetrics. The general experimental plan followed in this study is given in Table 2. A total of 96 laboratory experiments were conducted for the balanced mix design portion of this study. Several additional samples were also prepared and tested for the G_mm_ measurement and volumetric design stages.

## 3. Results and Discussion

Test specimens were prepared from the three selected mixes (see Table 1) with the target air-void content of 7% (except for Mix1_AV5). Binder contents from the volumetric design are given in Table 1. For Mix1 and Mix3, three different asphalt contents (AC) were used for balanced mix design: AC_design_ from volumetric mix design, AC_design_ – 0.5%, and AC_design_ + 0.5%. For Mix2, AC_design_ – 0.5% was too low and could result in a very dry mix (due to high RAP content) and, hence, the three asphalt contents considered were: AC_design_ from volumetric mix design, AC_design_ + 0.5%, and AC_design_ + 1%.

### 3.1. SCB Test Results

**Figure 2:**
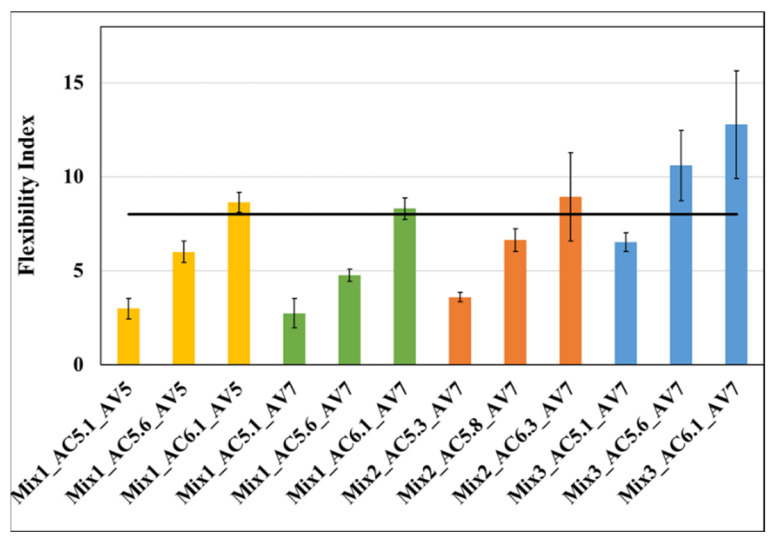
FI test results for all mixtures at 25 °C (length of the error bar is equal to one standard deviation). The number after AC indicates the percent asphalt (or binder) content. The number after AV represents the percent air voids of the samples.

The comparison of the cracking (SCB) performance of all asphalt mixtures along with their BMD variants is presented in Figure 2. The Flexibility Index (FI) parameter was used for this comparison. The horizontal black line in Figure 2 is the FI threshold selected in this study for Level 4 (FI_threshold_ = 8) mixtures as determined by Coleri et al. [11]. 

It can be observed from Figure 2 that the higher the binder content, the higher the FI for all cases. Thus, the FI parameter validated that increasing the binder content improves the cracking resistance of the mix. It should be noted that all the three mixes were Level 4 (highest traffic highway) mixtures.

From Figure 2, it can be observed that the average FI values of Mix3 were significantly higher than those of the other mixes. In Figure 2, the first bar for Mix2 and the second bar for the other mixes showed the FI value for the LMLC samples prepared at the volumetric design binder content. It can be observed that Mix3 had cracking resistances significantly higher than all other mixtures, which is likely to be a result of the use of a warm mix additive. It is important to mention that the mixtures with warm mix additive showed a better cracking resistance than other corresponding mixes with the same or higher binder contents. The FI value for Mix1 with 5% air-void was slightly higher than the same mix with 7% air void. Thus, the density of the mix appeared to have an effect on the cracking resistance. The high-RAP mix (Mix2) had a better cracking resistance than the low-RAP mix (Mix1), but this can be explained by the higher binder content of Mix2 specimens. BMD suggested the optimum binder contents (calculated and presented later in the article) for 30% and 45% RAP cases should be checked to determine the impact of increased RAP percentage on performance and design binder content.

### 3.2. HWTT Test Results

**Figure 3:**
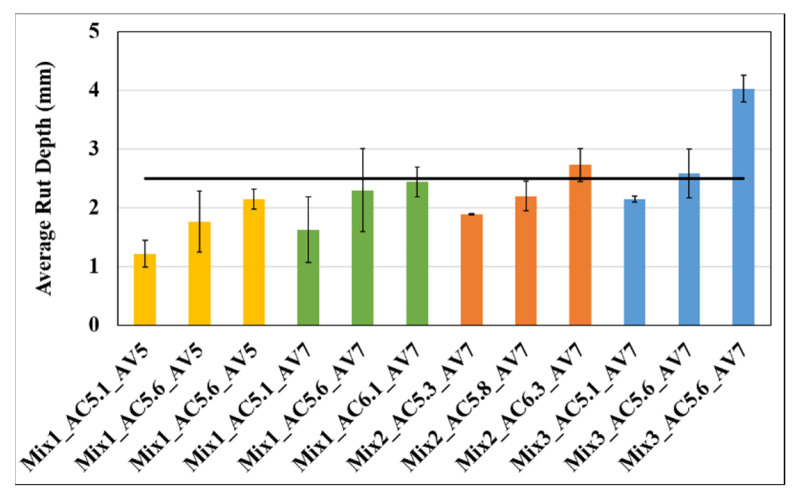
HWTT test results for all mixtures at 50 °C (length of the error bar is equal to one standard deviation). The number after AC indicates the percent asphalt (or binder) content and the number after AV represents the percent air voids of the samples.

Figure 3 presents the HWTT test results for all asphalt mixtures. Testing was conducted at 50 °C, and 20,000-wheel passes were used to determine a pair of samples’ average surface rut depth. The horizontal black line in Figure 3 is the HWTT rut depth threshold used in this study for BMD (RD_threshold_ = 2.5 mm for Level 4 mixes determined by Coleri et al. [11]). In this study, thresholds for FI and rut depth (RD) were selected to create reasonable binder content intervals. At the field implementation stage, these thresholds will be modified within the first years based on the laboratory- and field-measured rutting and cracking performance. To avoid any early rutting failures that might discourage the implementation of the BMD method, the RD threshold was selected to be a lower value (2.5 mm). This threshold will be gradually increased at the field implementation stage (expected to start in early 2023) to increase the design binder content and improve cracking resistance.

A higher binder content expectedly resulted in a higher rut depth for all the cases, as observed in Figure 3. In addition, it can be observed that Mix1_AV5 had the best rutting resistance among all the mixes. Samples for only this mixture were compacted at 5% air-void. A higher density (2% higher than the 7% air-void samples) resulted in an improved rutting resistance. It is important to note that a 2% increase in density resulted in significant improvements in both rutting and cracking performance. Although not simulated in this study, increased density is also expected to reduce the long-term aging and moisture susceptibility of the asphalt mixtures due to reduced permeability. It is possible that Mix3 with warm-mix additives can have a better “compactibility” due to the lower viscosity of the modified asphalt binder. Improved compactibility will result in higher density values with associated long-term performance benefits.

Mix3 showed the highest rut-depth among all three mixes as the warm mix additive and reduced aging during the mix preparation due to the lower mixing temperature, making the mix softer. The high-RAP mix showed a higher rut depth than the low-RAP mix (Mix1), but it should be noted that the high-RAP mix also had a higher binder content (0.2% more binder for every case).

The average rut depths of all the mixtures were lower than 3.0 mm (except the WMA mix with the highest binder content), which is significantly lower than the maximum rut depth allowed by several agencies in the U.S. (which is 12.5 mm). This result suggested that all mixes used in this part of the study are on the “dry” side and need more asphalt binder to improve their cracking resistance while still meeting the 12.5 mm maximum rut depth requirement.

### 3.3. Balanced Mix Design

**Figure 4:**
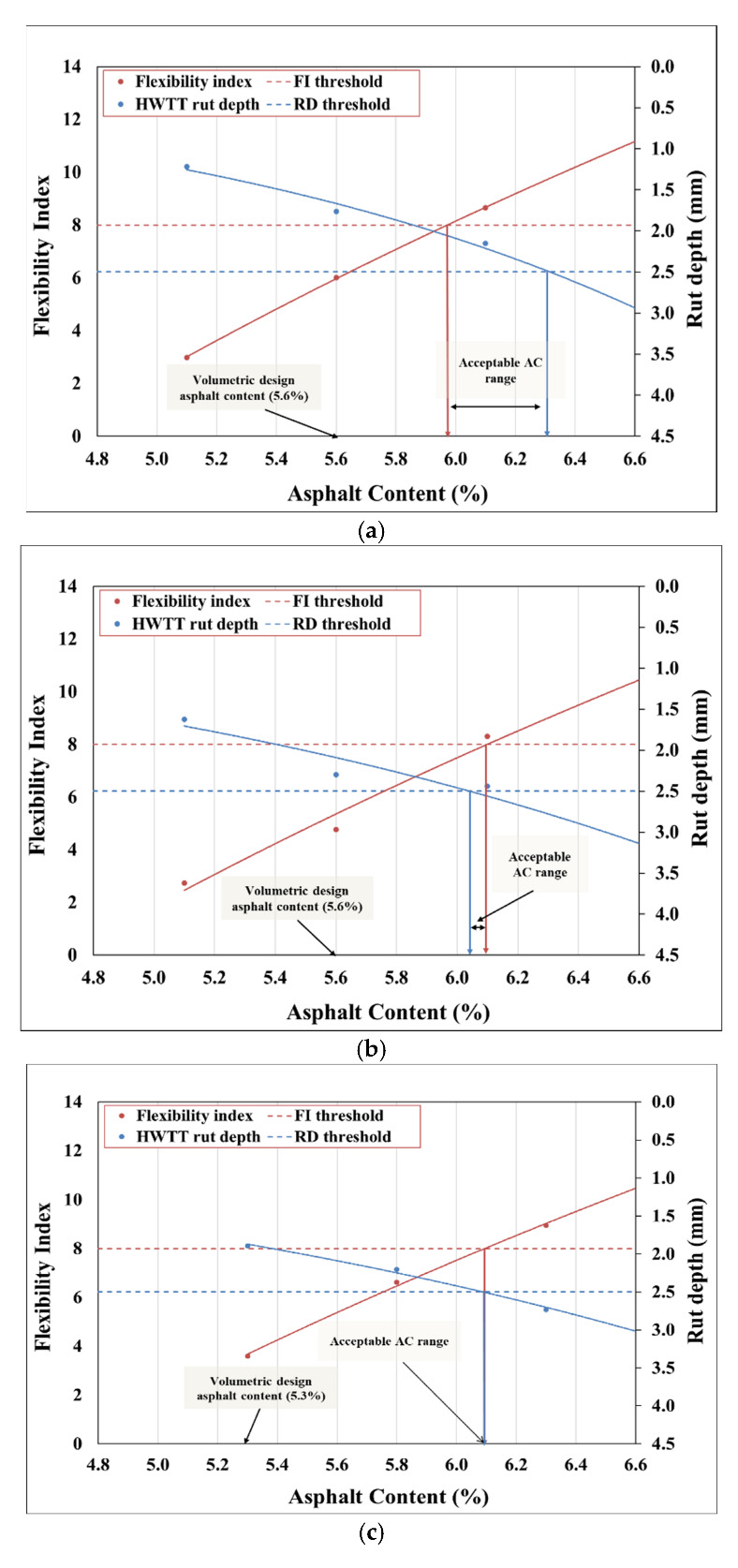

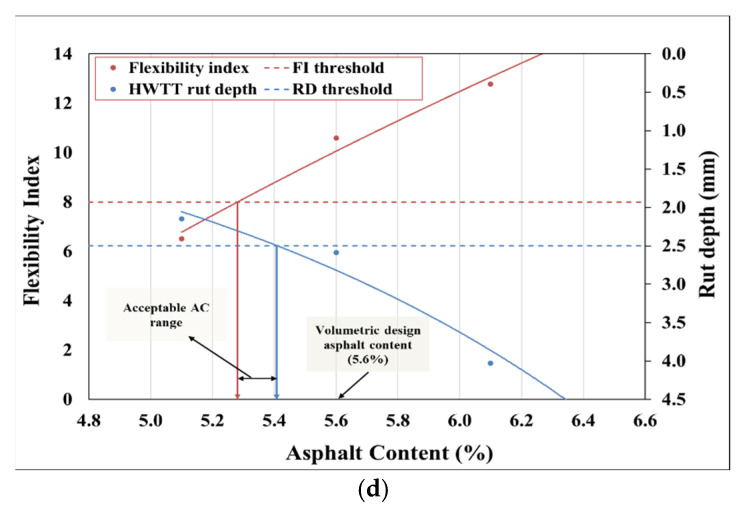
Balanced mix design for (**a**) Mix1_AV5, (**b**) Mix1_AV7, (**c**) Mix2, and (**d**) Mix3.

The balanced mix design concept is utilized to determine the maximum and the minimum asphalt content that “balances” the rutting and cracking performance of the asphalt mixtures. In Oregon, Coleri et al. [11] recommended a FI of 8 for Level 4 mixtures and FI of 6 for Level 3 mixtures as thresholds to be met by the minimum binder content allowed in the mix. A rut depth of 2.5 mm for Level 4 mixtures and 3 mm for Level 3 mixtures were the recommended ceilings for the allowable maximum binder content in the mix in Oregon [11]. Figure 4a–d depict balanced mix design charts for all the mixes used in this study. Based on the volumetric mix design, Mix1 and Mix3 have an asphalt content of 5.6%, and Mix2 has an asphalt content of 5.3%.

From Figure 4a, it can be observed that Mix1 does not meet the cracking and rutting criteria at the design asphalt content. However, with the balanced mix design approach, the minimum asphalt binder content required is about 6% (see Figure 4a). This increased binder content is expected to significantly increase the cost of the Mix1_AV5 asphalt mixture while still keeping it in the acceptable region for rutting and cracking performance. To ensure a high long-term cracking performance, a 6.3% asphalt binder content can also be used for production. However, it should be noted that using a 6.3% design asphalt content creates a high risk for rutting as plant-produced mixtures are allowed to have a ±0.5% variability in production binder content in Oregon. The ODOT is currently in the process of changing the binder content variability tolerance from ±0.5% to ±0.35%. This change is expected to reduce the risk of rutting or cracking failures due to production binder content variability. However, for practicality and considering the mix costs, this study recommends using the lower limit obtained from the balanced mix design approach. Similarly, based on the balanced mix design plots for the other three mixes, the required asphalt contents for Mix1_AV7, Mix2, and Mix3 are 6.05%, 6.10%, and 5.30%, respectively. Although there is no binder content range for Mix1_AV7 (See Figure 4b) that satisfies both the rutting and cracking requirements, the upper limit number that satisfies the rutting requirement is selected as the design binder content for balanced mix design.

### 3.4. Cost Comparisons

**Figure 5:**
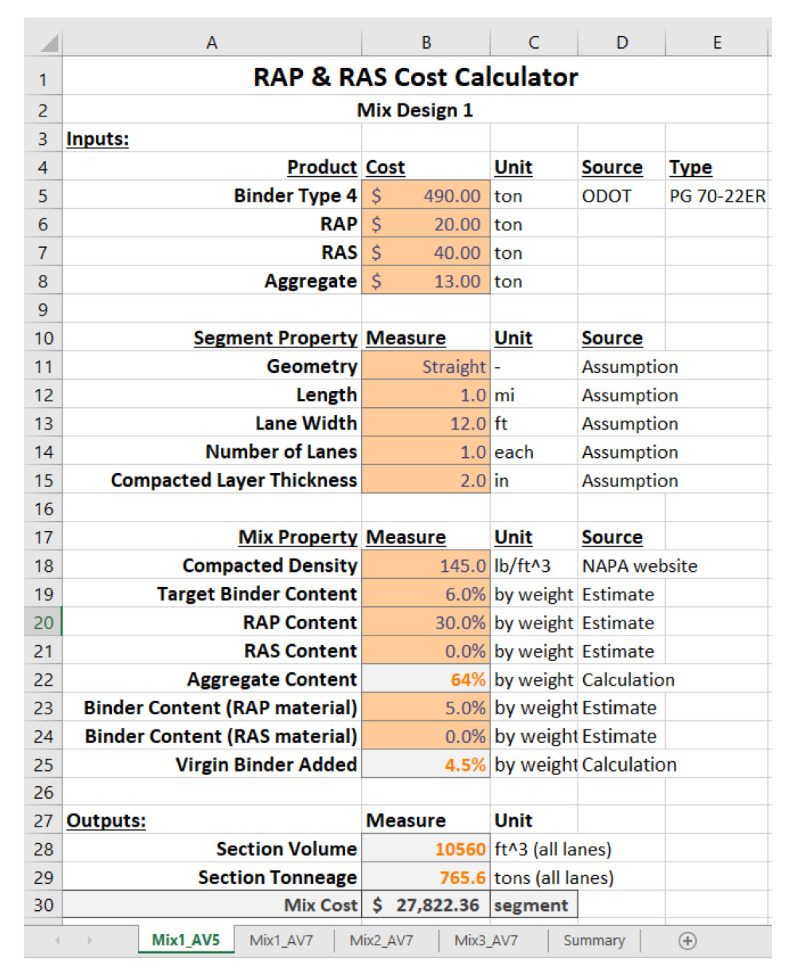
Cost calculation tool input tab.

In this study, a cost calculation tool created by Coleri et al. [25] was used to compare the costs and potential savings of different mix design strategies. A screenshot of the tool’s input tab is given in Figure 5. This tool requires the user to input data about the mix design, pavement section geometry, and costs of raw materials and provides an output in terms of weight, volume, and total cost of the mix. Further details and underlying assumptions of the tool can be found in the ODOT Research Project SPR797 [25]. The total material cost of asphalt mixtures was calculated using the following costs obtained from local providers for a previous study [25]:

- RAP: USD 20/ton;
- Aggregate: USD 13/ton;
- PG70-22ER binder: USD 490/ton;
- WMA additive: USD 70/ton (added to the per ton cost of the binder for a 0.7% WMA by weight of binder).

An additional step of calculating the production burner cost, which was not included in the original cost calculation tool, was incorporated in this study. The burner fuel cost can be the key factor in determining whether the HMA or the WMA is the most cost-efficient asphalt mixture. In order to assess the contribution of the production costs, a fuel consumption of 2 gallons of diesel fuel per ton for HMA and 1 gallon (3.78 L) of diesel fuel per ton for WMA with chemical additive [32] were considered, which means a reduction of 50% burner fuel. In addition, a price of USD 3/gallon diesel fuel for Oregon was used [33].

**Figure 6:**
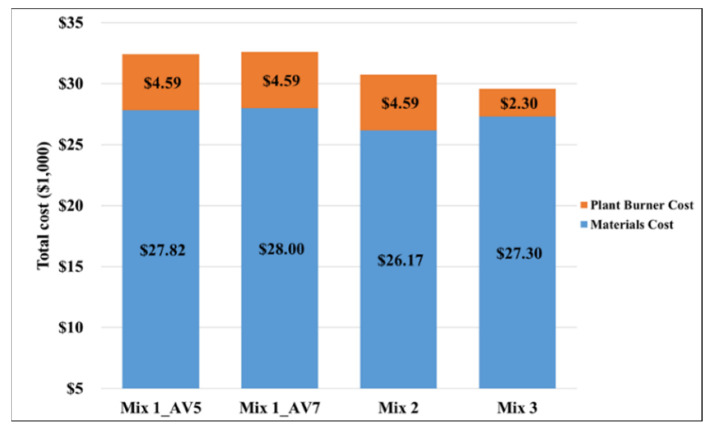
Cost comparison for all the mixes based on materials and burner fuel cost (costs calculated for a 1.61 km roadway section with a single 3.7 m wide lane and 50.8 mm of compacted asphalt concrete layer thickness).

Figure 6 presents the comparisons of all the mixes based on materials and plant burner fuel costs. It should be noted that the calculated asphalt mixture costs are based on the cost calculations in the spreadsheet by using the raw material costs and do not include any plant operation costs or added profit for the producer. As 45% RAP is not allowed in Oregon and warm-mix is not commonly used, it was not possible to obtain exact mixture costs for those alternatives.

### 3.5. Life-Cycle Cost Analysis

As a first step, life-cycle cost analyses were carried out on different mixes used in this study to compare the impact of the basic constituents of the HMA mix on life-cycle costs. Then, a second set of LCCA was performed after including the plant burner costs to be able to determine the cost impact of using warm-mix. The material costs were based on the cost calculation tool described earlier. The following assumptions were made for the pavement geometry:

- Lane—single;
- Width—3.7 m;
- Length—1.61 km.

A 4-percent interest rate was used to arrive at the Net present value (NPV) of agency costs for a 60-year analysis period by using Equation (2):

(2) $$\mathrm{NPV}=\sum_{t=0}^{T}\frac{C_{t}}{{\left(1+r\right)}^{t}}$$

where:

C_t_ = estimated agency costs at year t;
r = interest rate;
T = number of time periods.

As all mix designs had a 20-year design period, it was assumed that the same mixtures would be used every 20 years for the next 60 years. In this study, the NPV was calculated for all the mixes, and the equation below describes how the NPV for Mix1_AV5 was calculated (as an example).

$${\mathrm{NPV}}_{6\%\mathrm{BC}}\mathrm{Mix}1\_\mathrm{AV}5=\frac{\$27,823\;}{{\left(1+0.04\right)}^{0}}+\frac{\$27,823\;}{{\left(1+0.04\right)}^{20}}+\frac{\$27,823\;}{{\left(1+0.04\right)}^{40}}=\$46,316$$

**Figure 7:**
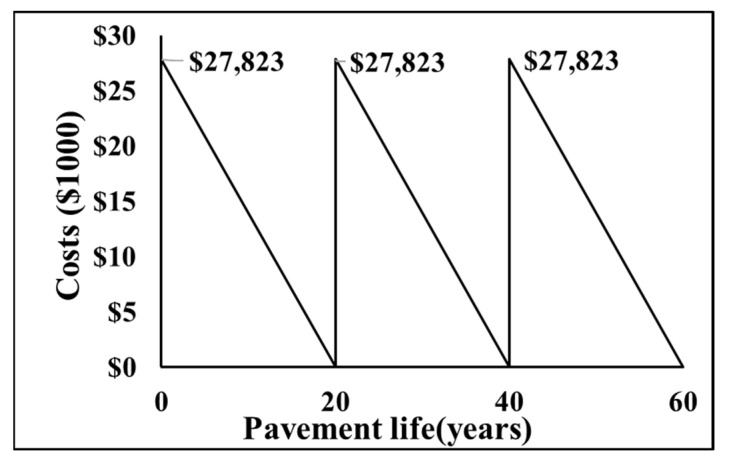
Diagram used for LCCA of Mix1_AV5.

Figure 7 provides an example diagram used for LCCA.

In Table 3, the NPVs without the burner fuel consumption costs (by just considering raw material costs) and the NPVs with the burner fuel consumption costs are summarized for all asphalt mixtures of this study.

It can be observed from Table 3 that the mix with 45% RAP content (Mix2) had the lowest NPV over the course of the 60-year analysis period, followed by the warm mix asphalt (Mix3) and then the mix with 30% RAP (Mix1) when only the raw material costs are considered. However, this ranking altered when the plant burner fuel consumption was incorporated into the life cycle cost analysis. When the burner costs are included in the LCCA, the most cost-effective mix is the warm mix asphalt (Mix3), considering the reduced production (burner) temperature and, consequently, less fuel consumption during production.

### 3.6. Environmental Impact Analysis

Pavement Life Cycle Assessment (LCA) procedures were used to calculate the environmental impact of each pavement mixture for the material production and construction stages of the pavement life cycle. In this study, a “cradle to gate” approach with the software’s pre-defined system boundaries was adopted. The functional unit was input as roadway parameters. The software utilizes proprietary databases on materials, energy, and transportation and reports a detailed set of life cycle impact assessment results. Moreover, the software supports the United States Environmental Protection Agency (US EPA) Tool for Reduction and Assessment of Chemical and Other Environmental Impacts (TRACI)-based midpoint life cycle impact assessment (LCIA) indicators: global warming potential, acidification potential, eutrophication potential, human health particulate, smog potential, ozone depletion potential, total primary energy consumption, non-renewable energy consumption, and fossil fuel consumption. Three of these indicators were selected for the LCA study as discussed later in the paper. 

For a base case, a mixture of 6% binder content and 20% RAP content was selected (referred to as Mix F in the plots). This represents the most common pavement design in Oregon. The roadway geometry for all cases was defined to have three lifts of pavement with thicknesses of 63.5 mm, 139.7 mm, and 139.7 mm. The length of the roadway was set to 1 km, with three lanes of 3.7 m each, a typical width for roadways in the U.S.

In order to determine the differences in environmental performance, the primary characteristics for each pavement design were entered into the Pavement LCA software. Materials by the percentage of total mixture weight were input (binder content, additives, RAP content, etc.) along with the asphalt type (HMA or WMA). All factors for which no data were available or factors that were not considered (such as hauling distance) were set to be default and equal between mixes to not affect the results. Pavement–vehicle interaction (PVI), which is a separate option in the software, was excluded entirely as all mixes were designed for 20 years, and PVI-related vehicle operating costs should be theoretically equal for all analyzed mixtures.

In order to accurately compare different pavement designs, each mixture was assumed to conform to a 60-year lifespan, with rehabilitation occurring every 20th year. For rehabilitation, 50.8 mm of asphalt is milled and removed and then replaced (mill and fill process, which is commonly used in Oregon for rehabilitation).

Results were exported from the software and plotted. Results are given in Global Warming Potential (GWP), Acidification Potential (AP), and Eutrophication Potential (EP) for all three mixtures of this study. Mix1 with the 5% air void case was not evaluated, as density does not directly change the environmental impact during the material production and construction stages. Units do not represent the chemical composition of the pollution itself but instead represent the amount of a standard normalizing factor representative of each pollution type [34].

**Figure 8:**
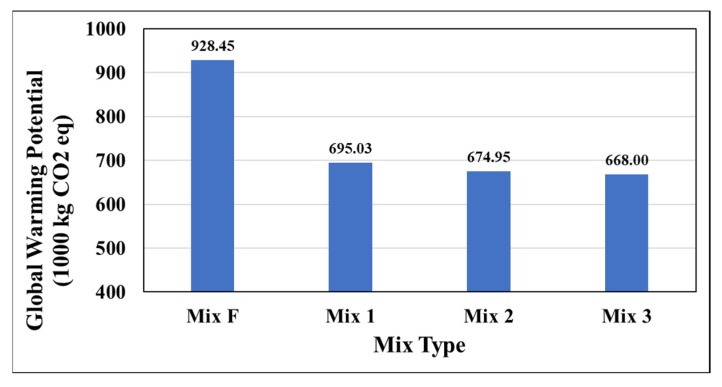
Global Warming Potential (GWP) by mix type.

Figure 8 displays the results for global warming potential by mix type, in units of kilograms of carbon dioxide. Global warming potential acts as a useful parameter to assess the future impact of emissions on the atmosphere [34].

Mixtures 1, 2, and 3 each performed nearly equivalently, with Mix1 exhibiting slightly worse performance and Mix3 (warm-mix) being the best. All mixtures had a significantly lower impact when compared to the typical Oregon asphalt mixture with lower RAP content. This is likely caused by the difference in the production process between HMA and WMA and higher RAP content in the designed mixtures. This result also proves the importance of further increasing the RAP content of asphalt mixtures for the environment. 

**Figure 9:**
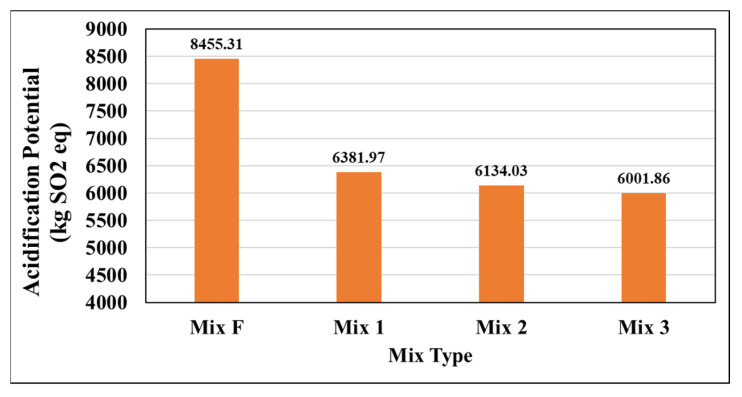
Acidification Potential (AP) by mix type.

Figure 9 displays the acidification potential of each pavement mix. Acidification results from carbon dioxide released into the atmosphere dissolving into ocean waters, which increases the concentration of carbonate ions and lowers ocean water pH [35].

The results for acidification potential were similar to those of global warming potential. Mix F again performed poorly, while Mixes 1, 2, and 3 performed similarly. Mix3 (warm-mix) again outperformed both Mixes 1 and 2. This is most likely a result of the WMA production process being significantly less energy-intensive, as well as the design allowing for a lower binder content and higher RAP.

**Figure 10:**
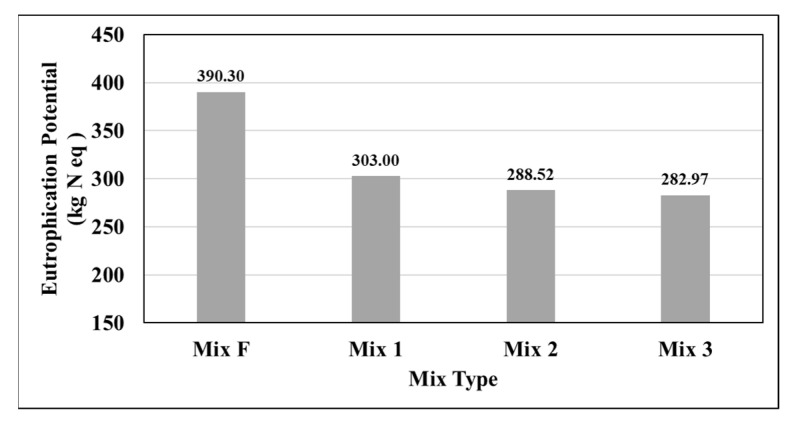
Eutrophication Potential (EP) by mix type.

Figure 10 displays the eutrophication potential generated by each mixture measured in kilograms of nitrogen. Eutrophication is a measure of the increased availability of normally population-limiting factors for aquatic-based photosynthetic organisms [36]. Increased eutrophication can lead to the destabilization of ocean ecosystems.

The results indicate that mixtures 1, 2, and 3 again outperformed the typical pavement design. Mix3 (warm-mix) performed the highest of the three design mixtures. The differences between the three design mixtures and the typical mixture are likely explained by the increased RAP content in the three designs, as well as the lower energy cost of WMA. Differences between the three designs are likely to be caused by the slight difference in binder content and RAP content.

## 4. Conclusions

In this study, volumetric and balanced mix designs were conducted to determine the optimum asphalt binder content for three different asphalt mixtures. Cost-effectiveness and the environmental impact of those asphalt mixtures were also quantified and compared. Based on the quantified cost, performance, and environmental impact values, the mixture with warm-mix additives (Mix3) was selected as the most preferable asphalt mixture with the highest cracking resistance, lowest cost, and lowest environmental impact. Other conclusions derived from this study are as follows:

1. Mix3 has a cracking resistance significantly higher than all other mixtures. A higher cracking resistance for Mix3 is likely to be a result of the use of a warm mix additive. It is important to mention that the mixtures with warm mix additive show a better cracking resistance than other corresponding mixes with the same or higher binder contents.
2. The cracking resistance for Mix1 with 5% air-void is slightly higher than the same mix with 7% air void. Thus, the density of the mix appears to have a significant effect on the cracking resistance.
3. The high-RAP mix (Mix2) has a better cracking resistance than the low-RAP mix (Mix1) according to SCB test results, but this is expected to be a result of the higher binder content of Mix2 specimens. The higher BMD binder content of Mix2 (when compared to lower-RAP mix—Mix1) suggests that the performance of a high-RAP mixture can be improved by slightly increasing the binder content.
4. Although Mix2 (45% RAP) has a higher BMD binder content than Mix1 (30% RAP), it is still more cost-effective due to the increased use of recycled asphalt material in the mix.
5. Mix1_AV5 has the best rutting resistance among all the mixes. Samples for only this mixture are compacted at 5% air-void. A higher density (2% higher than 7% air-void samples) results in an improved rutting resistance. It is important to note that a 2% increase in density results in significant improvements in both rutting and cracking performance. Although not simulated in this study, increased density is also expected to reduce the long-term aging and moisture susceptibility of the asphalt mixtures due to reduced permeability.
6. The mix with 45% RAP content (Mix2) has the lowest NPV over the course of the 60-year analysis period, followed by the warm mix asphalt (Mix3) and then the mix with 30% RAP (Mix1) when only the raw material costs are considered. However, this ranking is altered when the plant burner fuel consumption is incorporated into the life cycle cost analysis. When the burner costs are included in the LCCA, the most cost-effective mix is the warm mix asphalt (Mix3), considering the reduced production (burner) temperature and, consequently, less fuel consumption during production.
7. Mix3 (warm-mix) is also the most environmentally friendly mix with lower expected GWP, EP, and AP values for a 60-year analysis period.

## Figures and Tables

**Table 1:** Mix design and volumetric properties for the three trial mixes.

ID ^1^	Binder Grade	RAP ^2^ (%)	AC_RAP_^3^	AC ^4^ (%)	Pbe ^5^ (%)	P200/Pbe ^6^ Ratio	Addi. ^7^
			(%)				
Mix1_AV5	PG 70-22ER	30		5.6	4.60	1.4	1% Li ^8^
Mix1_AV7		30		5.6	4.60	1.4	1% Li
Mix2		45	5.02	5.3	4.35	1.7	1% Li
Mix3		30		5.6	4.60	1.4	1% Li, 0.68% WMA ^9^

^1^ All mixtures had dense gradation and aggregates with a nominal maximum aggregate size of 12.5 mm. ^2^ Reclaimed asphalt pavement added by weight. ^3^ Asphalt content by mass present in reclaimed asphalt pavement. ^4^ Total asphalt content by weight from volumetric design. ^5^ Effective asphalt content present by weight in the total mix. ^6^ Dust-to-binder ratio in the mix (0.8–1.6 is the range). ^7^ Additive. ^8^ Hydrated lime. ^9^ Warm mix additive.

**Table 2:** Experimental plan for balanced mix design.

Mix ID ^1^	Test	Temperature (°C)	Asphalt Content (%)	Replicates	Total
Mix1_AV5	SCB	25.0	OBC ^2^, −0.5%, +0.5%	4	12
	HWTT	50.0		4	12
Mix1_AV7	SCB	25.0	OBC, −0.5%, +0.5%	4	12
	HWTT	50.0		4	12
Mix2	SCB	25.0	OBC, +0.5%, +1%	4	12
	HWTT	50.0		4	12
Mix3	SCB	25.0	OBC, −0.5%, +0.5%	4	12
	HWTT	50.0		4	12

^1^ LMLC samples from three trial mixes as described in Table 1. ^2^ Optimum binder content obtained from volumetric mix design.

**Table 3:** NPVs for all mixtures.

Mix ID	Initial Cost (USD)	NPV (USD)—Without Burner Fuel Consumption Cost	Initial Cost (USD)	NPV (USD)—With Burner Fuel Consumption Cost
Mix1_AV5	27,823	46,316	32,416	53,962
Mix1_AV7	28,005	46,619	32,599	54,267
Mix2	26,167	43,560	30,761	51,207
Mix3	27,299	45,444	29,597	49,269

## Data Availability

Not applicable.

## Abbreviations

The following abbreviations are used in this manuscript:

AASHTO	American association of state highway and transportation officials
AASHTO T 209-12	Standard method of test for theoretical maximum specific gravity and density of hot-mix asphalt
AASHTO T 312-12	Standard method of test for preparing and determining the density of asphalt mixture specimens by means of the Superpave gyratory compactor
AASHTO T 324	Standard method of test for Hamburg wheel-track testing of compacted asphalt mixtures
AC	Total asphalt content
ACRAP	Asphalt content by mass present in reclaimed asphalt pavement
AP	Acidification potential
BMD	Balanced mix design
DOT	Department of transportation
EP	Eutrophication potential
FI	Flexibility index
Gmm	Theoretical maximum specific gravity
GWP	Global warming potential
HMA	Hot mix asphalt
HWTT	Hamburg wheel tracking test
LCA	Life cycle assessment
LCCA	Life cycle cost analysis
LCIA	Life cycle impact assessment
LMLC	Laboratory mixed-laboratory compacted
LTA	Long-term aging
NPV	Net present value
OBC	Optimum binder content
ODOT	Oregon Department of Transportation
OSU-AMaP	Oregon State University Asphalt Materials and Pavements
P200	Percent aggregate passing No. 200 sieve
P200/Pbe	Dust-to-binder ratio
Pbe	Effective asphalt content
PMA	Polymer-modified asphalt
PVI	Pavement–vehicle interaction
RAM	Recycled asphalt materials
RAP	Reclaimed asphalt pavements
RD	Rut depth
SCB	Semi-circular bend
SGC	Superpave gyratory compactor
SHRP	Strategic highway research program
STA	Short-term aging
TRACI	Tool for Reduction and Assessment of Chemical and Other Environmental Impacts
USEPA	United States environmental protection agency
VFA	Voids filled with asphalt
VMA	Voids in mineral aggregates
WMA	Warm mix additive
